# Supplementary material for: The vascular gene Apold1 is dispensable for normal development but controls angiogenesis under pathological conditions
Source: Angiogenesis. 2023 Mar 18;26(3):385–407. doi: 10.1007/s10456-023-09870-z (PMC10328887; doi:10.1007/s10456-023-09870-z)
Supplement: Supplementary file 1 — Supplementary file1 (DOCX 1084 KB) [file 10456_2023_9870_MOESM1_ESM.docx]

**
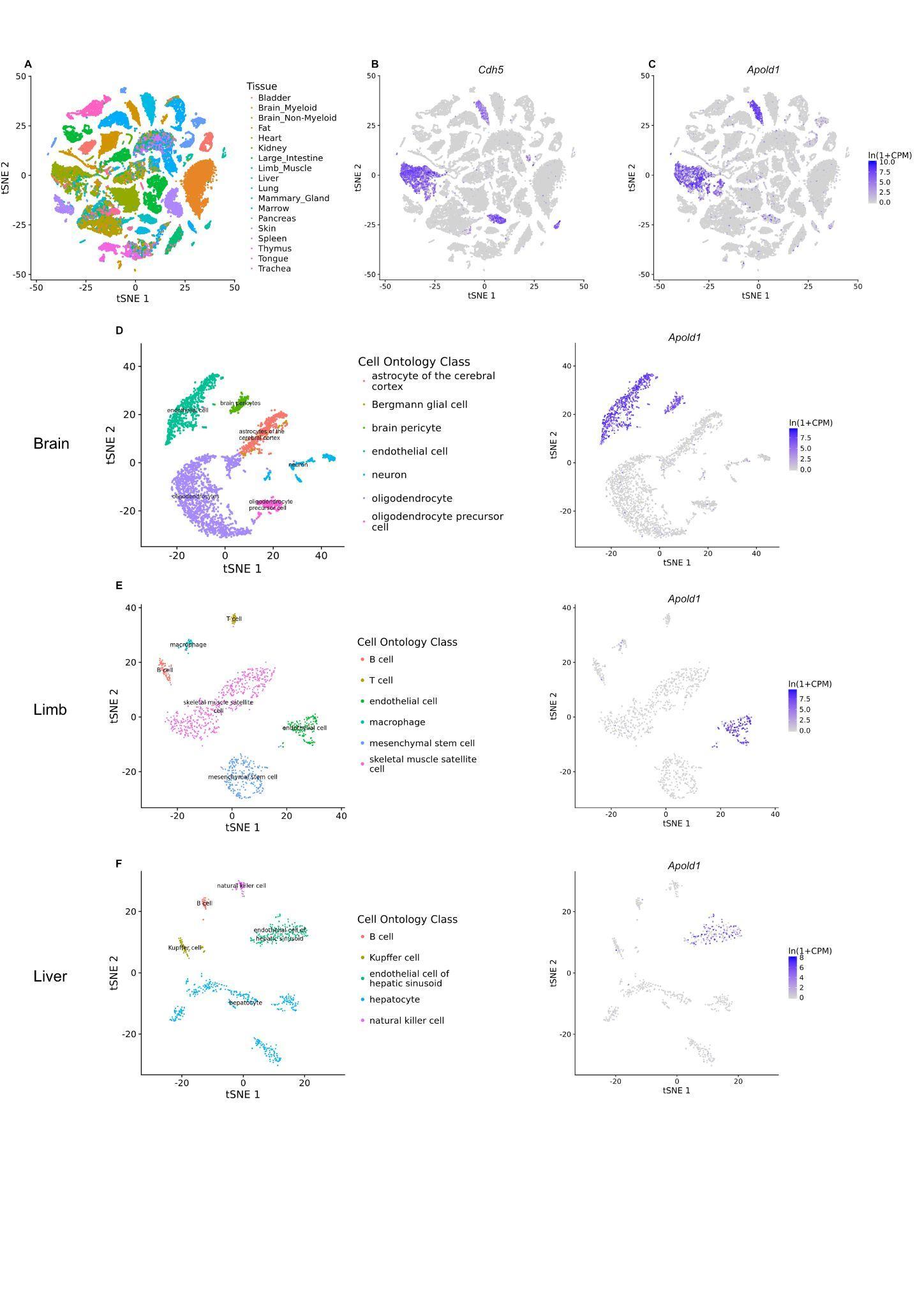
**

**Fig. S1 Across multiple organ systems in the mouse, *Apold1* expression is largely restricted to endothelial cells** (A) Analysis of the 'Tabula Muris' compendium of FACS sorted data across organs [54]. (B) Endothelial cells identified using *Cdh5* expression co-localize with (C) *Apold1* expression. (D) Across organs, endothelial cells in various tissues are highly enriched in *Apold1*. Restricting the 'Tabula Muris' dataset to FACS sorted cells, the *Apold1* enrichment in ECs is apparent in various highly vascularized tissues, including (E) the brain, (F) muscles, and (G) the liver.

**
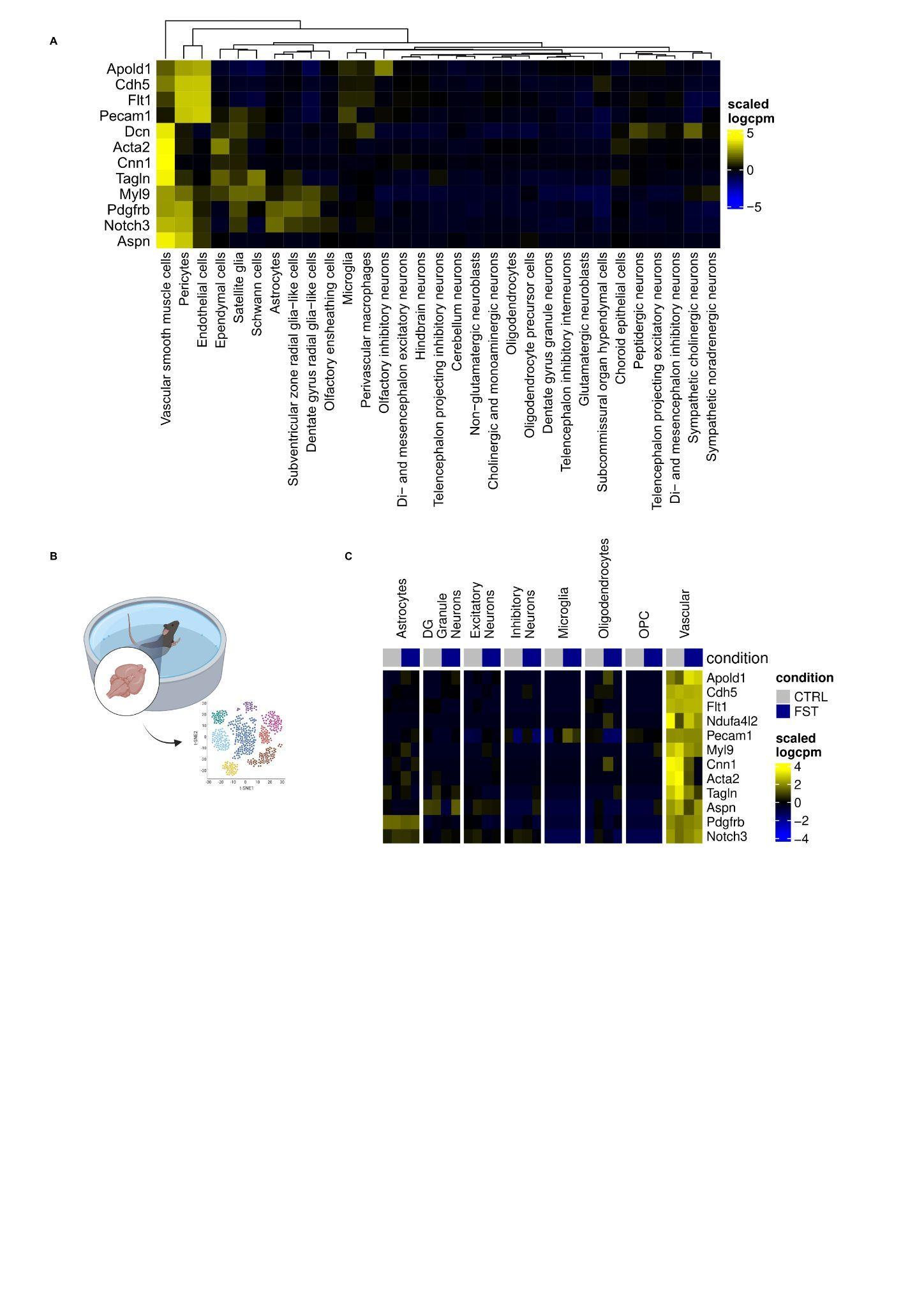
**

**Fig. S2 *Apold1* expression in the Zeisel et al 2018 study, an extensive single-cell characterization of the mouse nervous system** [45]

**
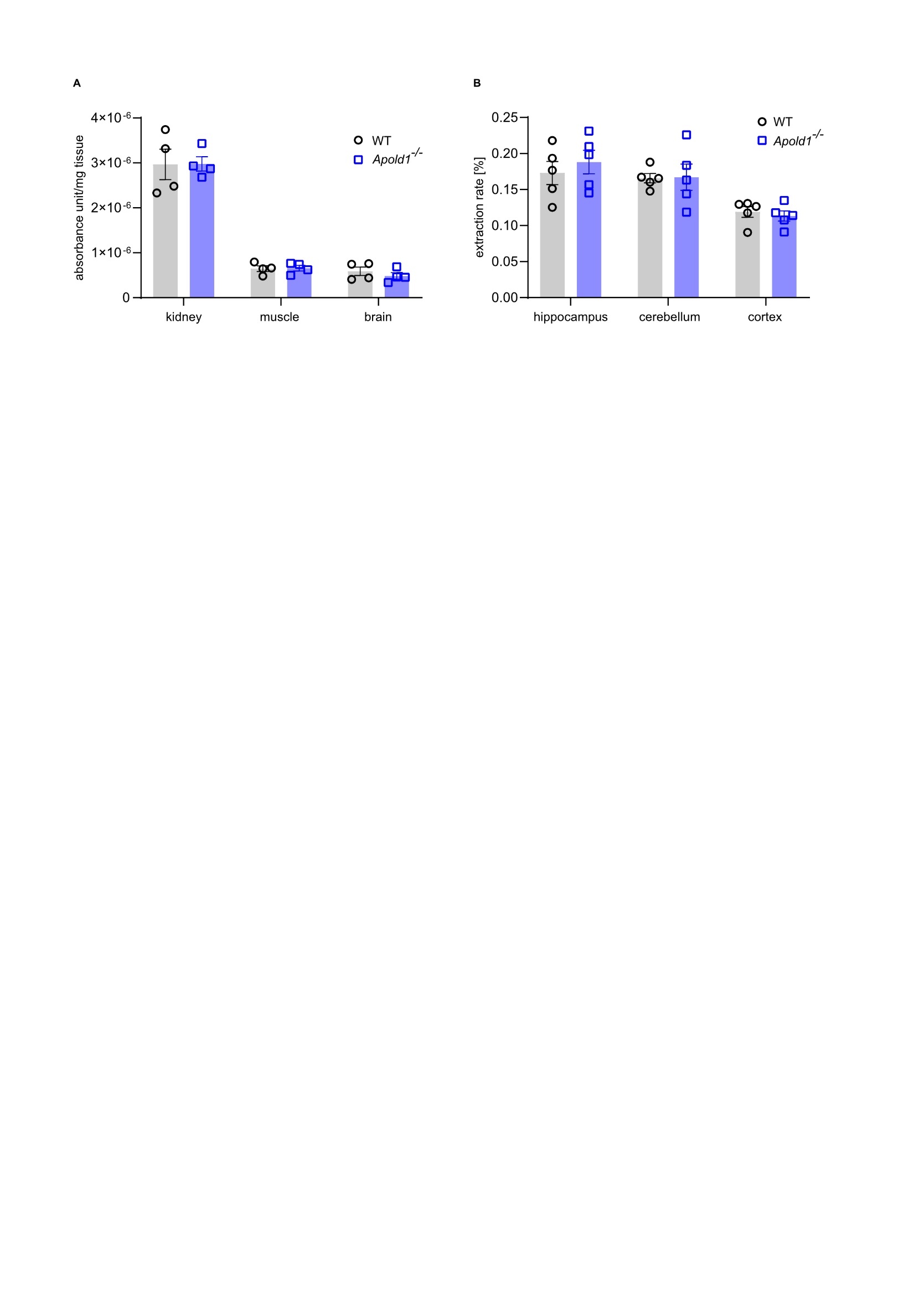
**

**Fig. S3. Apold1 deficiency does not affect vascular permeability in physiological conditions** (A) Vascular permeability measured by extravasation of Evans blue dye in kidney, muscle and brain (n (WT/*Apold1^-/-^*) = 4/4). (B) Blood-brain-barrier permeability assessed by extravasation of sodium fluorescein in the hippocampus, cerebellum and cortex (n (WT/*Apold1^-/-^*) = 5/5). The data shown are mean ± SEM.


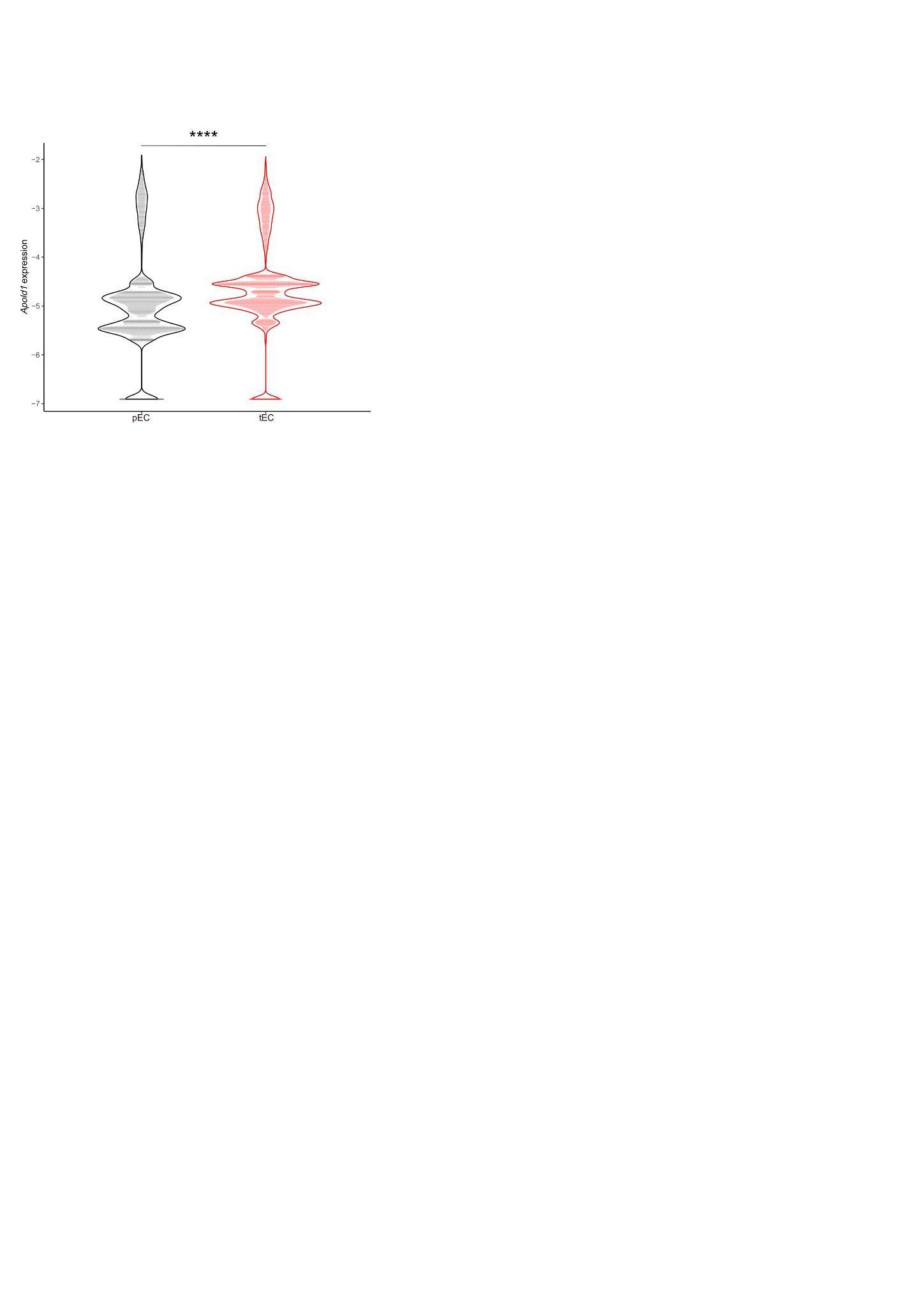


**Fig. S4** **High *Apold1* expression in tumor ECs and specifically in tip cells** scRNA-seq data reanalyzed [66]. Freshly isolated human tumor ECs (TECs) were analyzed in direct comparison to peritumoral pulmonary non-tumor ECs (PNECs) from the same patient. Data come from 1 large cell carcinoma, 4 squamous cell carcinomas, and 3 adenocarcinoma treatment-naive patients. (A) *Apold1* expression in TECs versus NECs (pEC mean= -4.9; tEC mean = -4.58; **** p < 0.0001).


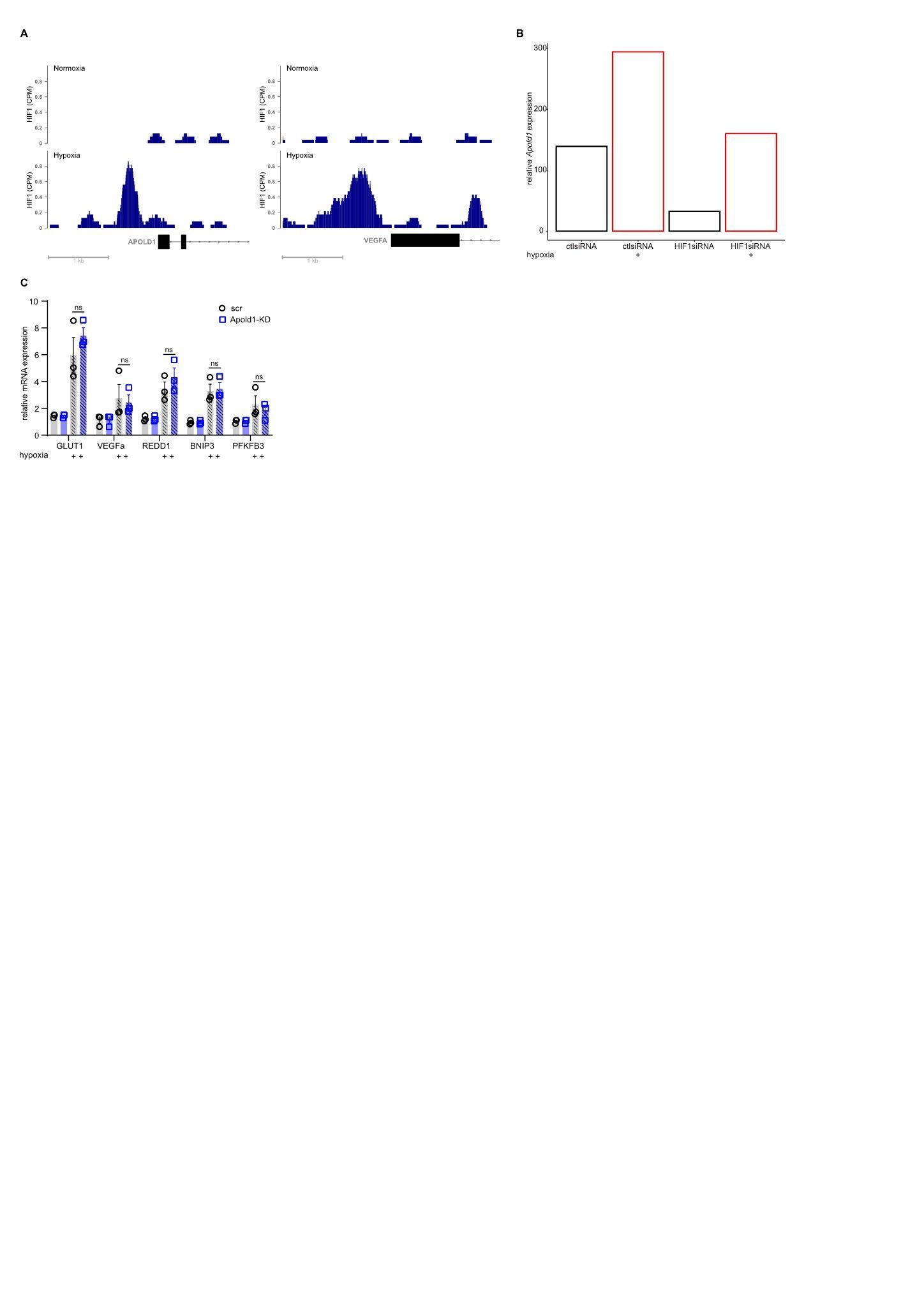


**Fig. S5 HIF1 knockdown prevents the hypoxia-induced increase in Apold1 in HUVECs** (A) Hypoxia dependent HIF binding sites to the promoter region of Apold1 and VEGF reanalyzed from a HIF1-ChIP-seq data-set [38]. (B) *Apold1* expression in HUVECS in response to Hif1a-knockdown under normoxia and hypoxia reanalyzed [38]. (C) RT-qPCR results for canonical hypoxia-responsive mRNAs in scr and Apold1-KD under normoxia (21% O_2_) and hypoxia (0.1% O_2_) for 16 hrs (n (scr/Apold1-KD) = 3/3). Student’s t-test in C. The data shown are mean ± SEM.


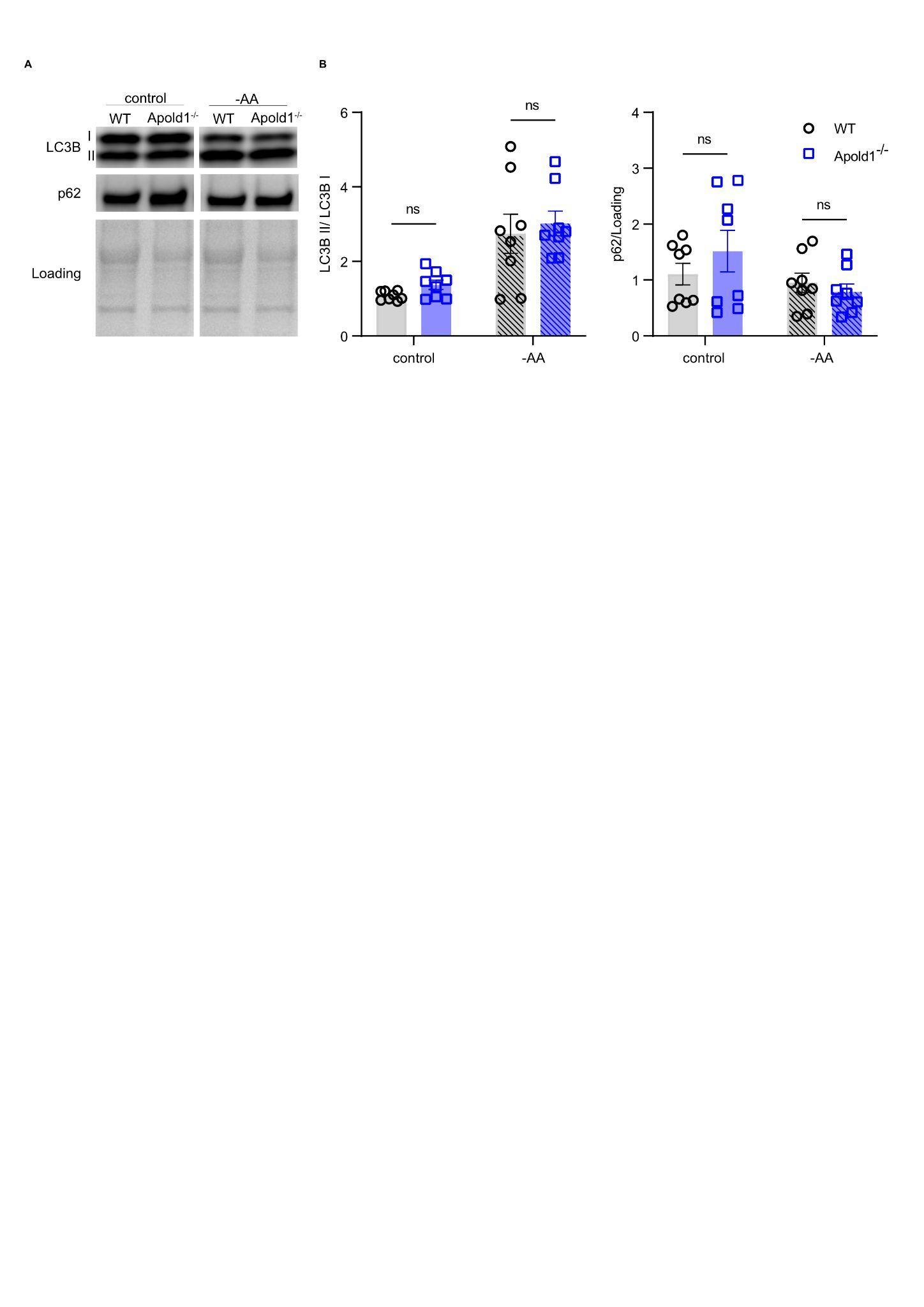


**Fig. S6 Apold1 deficiency is not affecting autophagic markers** (A) Representative images of western blot analysis of autophagic markers in control and amino acid (-AA) deprivation conditions in cultured WT and *Apold1^-/-^* mECs. (B) Quantification of LC3B ratio and p62 in control and amino acid deprivation (n (WT/*Apold1^-/-^*) = 8/8). Student’s t-test in B. The data shown are mean ± SEM.


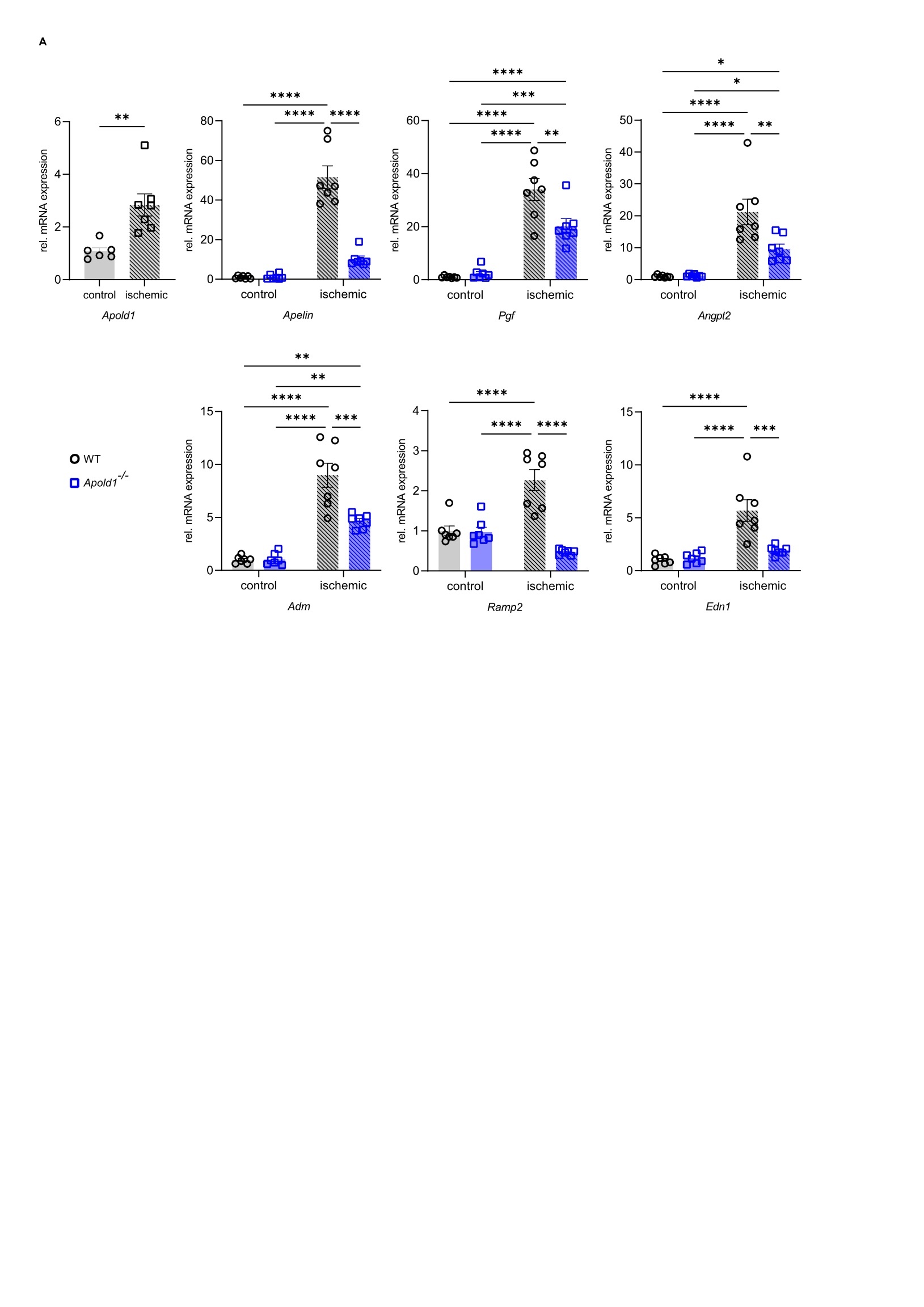


**Fig. S7 Various angiogenic genes show no differences under baseline conditions but are downregulated in *Apold1^-/-^* muscle after hindlimb ischemia** (A) Relative mRNA expression of angiogenic genes *Apold1*, *Apelin*, *Pgf*, *Angpt2*, *Adm*, *Ramp2* and *Edn1* in the soleus under baseline conditions and 24 hrs after hindlimb ischemia (n (WT/*Apold1^-/-^*) = 7/7). Student’s test in A left; Two-way ANOVA with Tukey’s multiple comparison test in A (*p<0.05; **p< 0.01; p<0.001; ****p< 0.0001). The data shown are mean ± SEM.
